# MAPPING BARRIERS AND FACILITATORS OF ACCESS TO HEARING HEALTHCARE RESOURCES TO THE COM-B FRAMEWORK

**DOI:** 10.1093/geroni/igac059.1386

**Published:** 2022-12-20

**Authors:** Debra Schutte, Thomas Templin, Brian Schutte

**Affiliations:** Wayne State University, Detroit, Michigan, United States; Wayne State University, Detroit, Michigan, United States; Michigan State University, East Lansing, Michigan, United States

## Abstract

Age-related hearing impairment (ARHI) affects 20-40% of older adults. However, many people with hearing impairment fail to seek professional evaluation and treatment, delay seeking treatment, or abandon treatment. The purpose of this study was to identify barriers and facilitators of access to hearing healthcare resources in a rural setting and to characterize these barriers within the COM-B (capability, motivation, and opportunity) framework. Survey packets were handed out following services at a rural community church; 72 completed surveys were returned. The mean age of participants was 54.4 years. Thirty-one percent of respondents reported moderate to severe hearing impairment; of these individuals, 7 (31%) were currently using a hearing aid. Rates of hearing protection use fell below the Healthy People 2020 target of 53% across occupational and leisure noise exposure. Forty-eight percent (N=34) of participants had never had their hearing tested as an adult. Overall perceived barriers were significantly correlated with age (r= .260, P = .034) and perceived hearing loss (r=-.277, P= .025). Barriers were identified across all domains and subdomains of the COM-B framework. Barriers clustered in the reflective motivation domain, in which participants undervalue the significance of hearing loss. Barriers also clustered within the social and physical opportunity domains, suggesting a lack of access to providers and an undervaluation of hearing loss by others (family and health care providers). These findings provide evidence regarding the accessibility of hearing healthcare services in rural settings and will serve as a foundation for the design of a community-tailored hearing health intervention.

